# Stronger diversity effects with increased environmental stress: A study of multitrophic interactions between oak, powdery mildew and ladybirds

**DOI:** 10.1371/journal.pone.0176104

**Published:** 2017-04-18

**Authors:** Mathias Dillen, Christian Smit, Martijn Buyse, Monica Höfte, Patrick De Clercq, Kris Verheyen

**Affiliations:** 1 Conservation Ecology group, Groningen Institute for Evolutionary Sciences (GELIFES), University of Groningen, Groningen, the Netherlands; 2 Forest & Nature Lab, Department of Forest and Water Management, Ghent University, Gontrode, Belgium; 3 Laboratory of Phytopathology, Department of Crop Protection, Ghent University, Ghent, Belgium; 4 Laboratory of Agrozoology, Department of Crop Protection, Ghent University,Ghent, Belgium; Università Politecnica delle Marche, ITALY

## Abstract

Recent research has suggested that increasing neighbourhood tree species diversity may mitigate the impact of pests or pathogens by supporting the activities of their natural enemies and/or reducing the density of available hosts. In this study, we attempted to assess these mechanisms in a multitrophic study system of young oak (*Quercus*), oak powdery mildew (PM, caused by *Erysiphe* spp.) and a mycophagous ladybird (*Psyllobora vigintiduopunctata*). We assessed ladybird mycophagy on oak PM in function of different neighbourhood tree species compositions. We also evaluated whether these species interactions were modulated by environmental conditions as suggested by the Stress Gradient Hypothesis. We adopted a complementary approach of a field experiment where we monitored oak saplings subjected to a reduced rainfall gradient in a young planted forest consisting of different tree species mixtures, as well as a lab experiment where we independently evaluated the effect of different watering treatments on PM infections and ladybird mycophagy. In the field experiment, we found effects of neighbourhood tree species richness on ladybird mycophagy becoming more positive as the target trees received less water. This effect was only found as weather conditions grew drier. In the lab experiment, we found a preference of ladybirds to graze on infected leaves from trees that received less water. We discuss potential mechanisms that might explain this preference, such as emissions of volatile leaf chemicals. Our results are in line with the expectations of the Natural Enemies Hypothesis and support the hypothesis that biodiversity effects become stronger with increased environmental stress.

## Introduction

In the last two decades, there have been an increasing number of studies concerning the influence of species composition on the functioning of ecosystems [1–3]. Positive effects of increasing diversity were found in many different systems, including forests, and for many different ecosystem functions, including biomass production [4] and mitigated impacts of pests and pathogens–also known as associational resistance [5,6]. Many mechanisms have been proposed to explain these community effects, including host dilution for specialist pests [7], complementarity of resource use [8] and direct facilitation such as through fertilizing effects of legumes [9]. Research has also suggested an important role of multitrophic interactions through natural enemies of pests [10]. According to the Natural Enemies Hypothesis, predators may diversify their diet in more diverse stands nutritionally as well as temporally and have access to more diverse habitats for shelters or oviposition [5,11]. However, questions remain concerning the role of the ecological traits of these natural enemies, as increasing species richness in a stand is often associated with increasing pest richness, which may impair the efficacy of specialist natural enemies such as most hyperparasites [12,13].

Oak powdery mildew (PM, caused by *Erysiphe* spp.) is the most important disease of West European oak (*Quercus*) species. As the disease is caused by a biotrophic fungal leaf pathogen, it may reduce net carbon assimilation as well as leaf lifespan and is associated with reduced radial growth or even increased mortality, especially in saplings [14–17]. First sighted in Europe in 1907 [18], the disease may play a role in the poor shade tolerance and regeneration of young oak saplings under canopy [19] and is the chief reason for frequent fungicide use on oaks in tree nurseries [15,20]. Recent research into the causal agents of oak PM differentiated at least four different species (*E*. *alphitoides*, *E*. *quercicola*, *E*. *hypophylla* and *Phyllactinia roboris*), of which *E*. *alphitoides* is the most frequently observed [21,22].

Grazing of powdery mildews by mycophagous ladybirds [23] has been observed across the world [24–28]. While several species of aphidophagous ladybird were found to include powdery mildews as a minor part of their diet [29], ladybirds of the tribe Halyziini are strongly suspected of being obligate mycophages, possibly even limited to only mildew species [24]. The ladybird *Psyllobora vigintiduopunctata* is a member of this tribe and occurs relatively abundantly in Western European woodland elements and young forest patches. Given that tree saplings will suffer the strongest impact of mildew infections [15], these ladybirds might play an important ecological role in mitigating the impact of PM infections. Furthermore, given that PM is caused by a specialist fungal pathogen and these ladybirds may be specialized feeders on mildew, this system could shed light on the relationship between the composition effect hypotheses of host dilution and natural enemies.

Effects of neighbourhood species composition might be dependent on environmental factors [13,30]. Recent research into the context dependency of neighbourhood species richness effects raised some evidence for the Stress Gradient Hypothesis, where positive interactions between species are more frequent in stressful conditions [31–33]. Therefore, if the Stress Gradient Hypothesis applies, a greater incidence of stressful conditions would increase the impact of neighbourhood composition effects, as more facilitative interactions will be expected. Such an interaction between plant stress and composition may have important ramifications in the light of Anthropogenic Global Change, which is expected to increase the frequency of stressful conditions such as drought or outbreaks of invasive pests [34]. Hence, it might become more important to increase diversity in species-poor systems or halt the global trend of biodiversity loss to preserve ecosystem functioning [2,35], as positive interactions would play a greater role under the changing conditions than expected from current assessments. While previous research found no great impact of dry weather conditions [15] or air humidity [36] on mildew infection rates, the response of mycophagous ladybirds to changing plant water conditions is not known. Changing water conditions may affect palatability of the fungus or the ability of the ladybird to find infected leaves by increased emission of signal molecules [37].

In this study, we set out to elucidate the impact of natural enemies on oak PM infections of oak (*Q*. *robur*) saplings in a young planted forest stand. We looked specifically at the ladybird *P*. *vigintiduopunctata*, due to its abundance in the study area, in particular on *Quercus*, and because previous studies already indicated that relatives of this species were capable of causing substantial reductions in infected leaf area through mildew grazing [24]. We hypothesized that increasing tree species richness had a mitigating effect on oak PM levels. Attempting to link composition effects to the Natural Enemies Hypothesis, we hypothesized that PM mycophagy and ladybird counts were similarly related to richness effects. To investigate the interplay between composition effects and the Stress Gradient Hypothesis, we established a rainfall reduction gradient for our focal saplings, predicting that effects of species richness on ladybird activity would be more important under more stressful conditions. Given the little knowledge available on the relationship between plant water status and mycophagy by ladybirds, we also conducted a lab study where the impact of neighbourhood species composition was eliminated. We specifically looked at the impact of different watering rates on PM infection severity and on the attractivity of mycelia for the ladybirds.

## Materials and methods

### Field experiment

**Fig 1:**
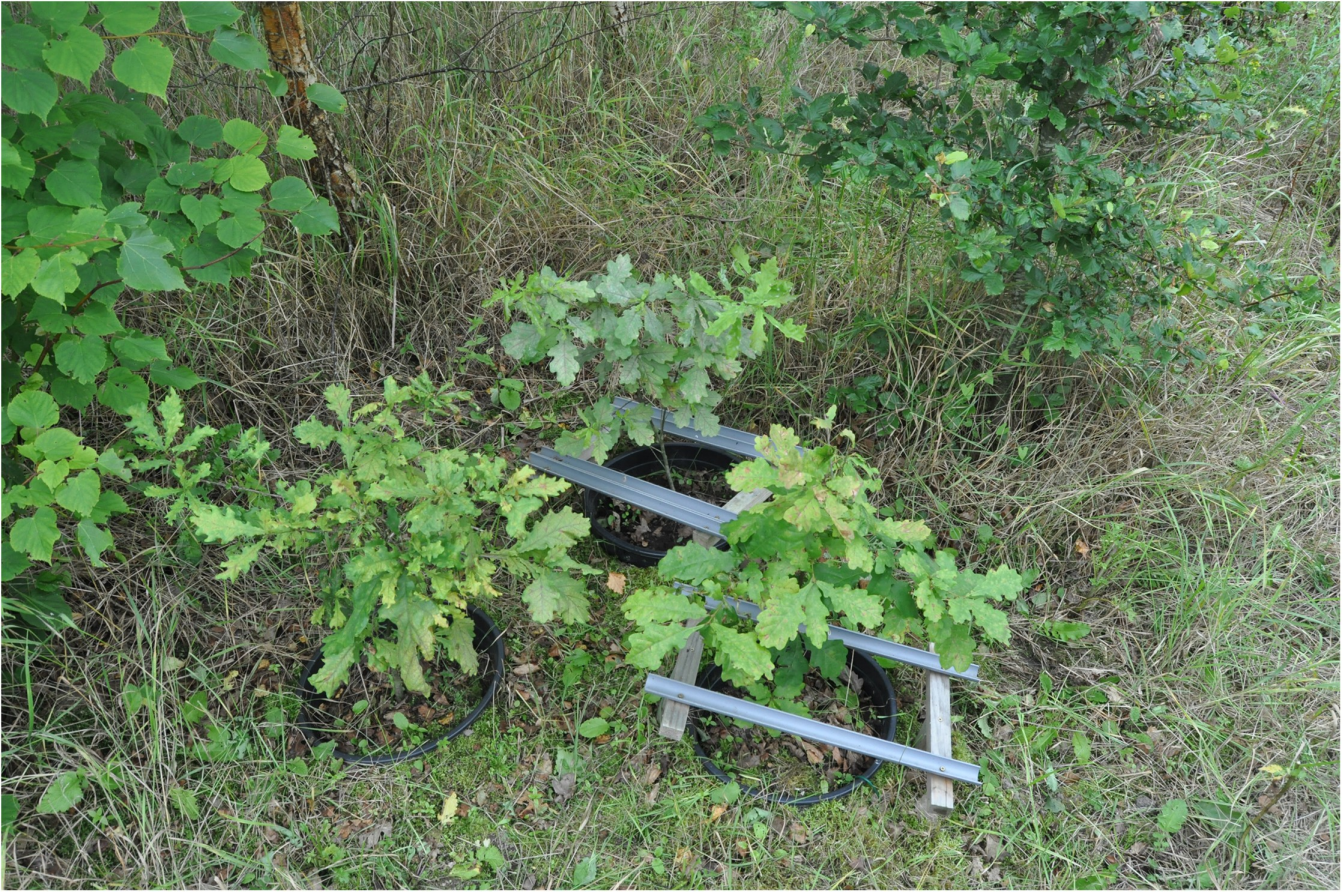
Example of a phytometer plot with the three rainfall reduction treatments. Three of the four surrounding FORBIO trees are visible.

Our experiment was conducted as part of a larger study into tree species composition effects and took place in Zedelgem, one of the three FORBIO sites in Belgium [38]. This site was planted in late 2009 and early 2010 with monocultures and mixtures of saplings of *Quercus robur*, *Fagus sylvatica*, *Pinus sylvestris*, *Tilia cordata* and *Betula pendula*, planted in groups of 3 by 3. In a total of 40 plots, 5 compositions for each species richness level from 1 to 4 were represented, including all five monocultures and 4-species mixtures; each composition was replicated once. In early May 2013, one-year old *Q*. *robur* saplings were planted in separate pots between 4 of the older FORBIO trees. Saplings were planted in groups of 3, each subjected to a different treatment of reduced rainfall by placing 0, 1 or 2 pairs of gutters above the pot surface but below the sapling canopy (Fig 1). A more extensive description of the experimental setup can be found in Dillen et al. [17].

**Fig 2:**
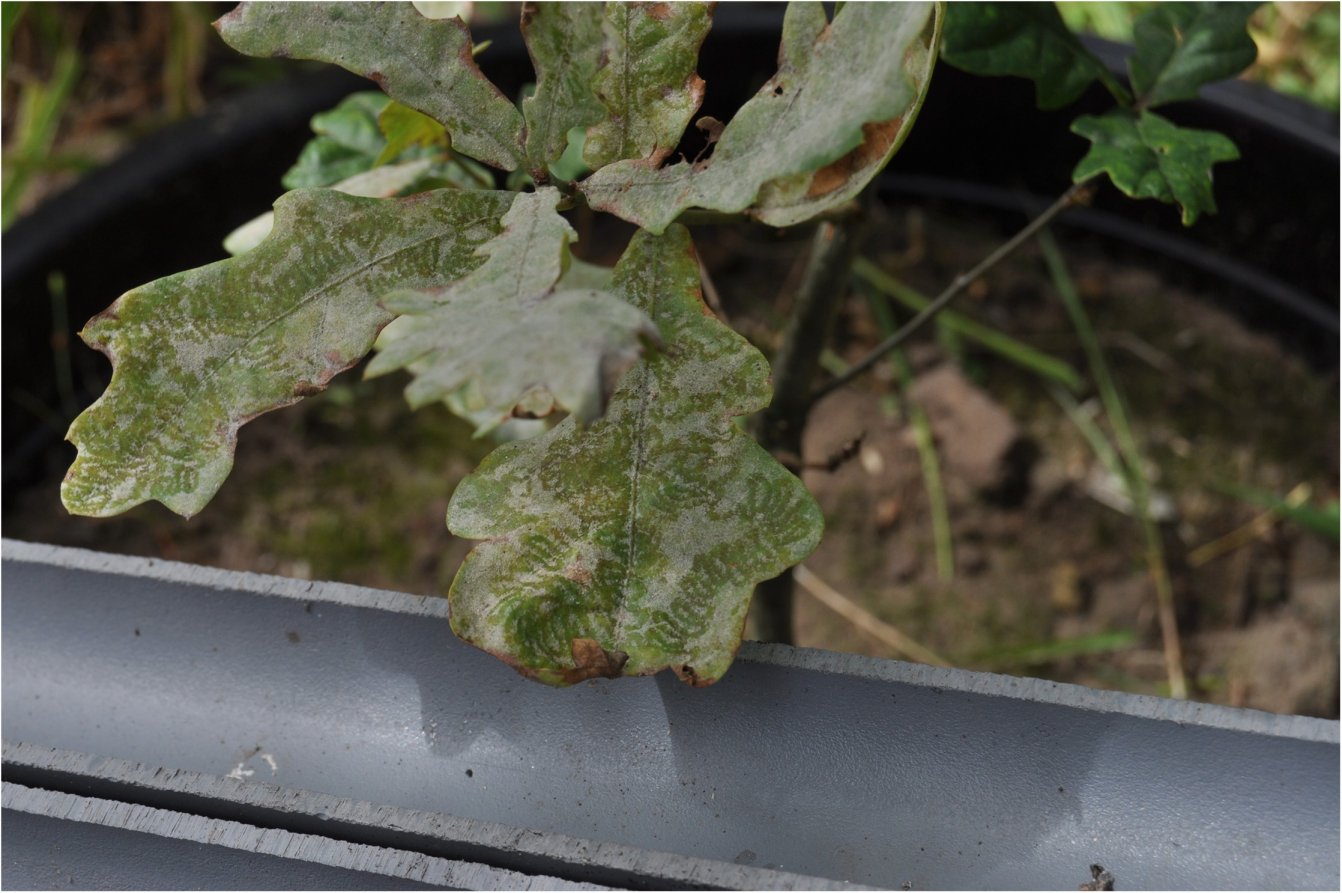
Example of ladybird grazing marks on oak PM.

In 20 of the Zedelgem plots, a monitoring for oak PM, PM mycophagy and mycophagous ladybird occurrence was performed bi-weekly four times in August and the first half of September 2014. All first order shoots were assessed using affected area classes of 0–5%, 6–30%, 31–60% and 61–100%. We also estimated a size weight for each shoot, indicative of relative differences in leaf area between first order shoots using the same approach as in Dillen et al. [17]. The PM scores included the PM grazing marks (Fig 2), so they could be used as a control variable for initial PM infected area. Mycophagy was assessed as % of total leaf area, not the area infected by PM. A weighted average of the class scores of the shoots was subsequently calculated to get PM infected leaf area percentages at the sapling level, using the shoot size weights and the class mids, except 0% for the smallest class (i.e. 0, 17.5, 45 and 80%). *P*. *vigintiduopunctata* occurrence was assessed by counting all present individuals that could be found on a sapling (in their 3^rd^ growing season at the time) in 1 minute. We differentiated between larvae, pupae and adults, but did not look for eggs as these are very small and therefore very difficult to find and identify.

All statistical analyses were performed using R [39]. For mixed models, we used the R package *lme4* [40], with *lmerTest* extension [41] to determine Satterthwaite approximated p-values. Testing the influence of composition, reduced rainfall (DR) and their interaction on PM infection levels was done using mixed models, with plot as a random factor. Models were run separately for the four monitoring moments, as weather conditions changed between them. Models were also run separately testing the influence of neighbourhood species richness (SR, model 1) and species identity (SP, model 2), quantified as species presence or absence.

PM(%)=SR+DR+SR*DR (1)

$$\mathrm{PM}(\%)=\sum_{i=1}^{5}SP_{i}+\mathrm{DR}+\sum_{i=1}^{5}SP_{i}*\;\mathrm{DR}$$ (2)

Where ***PM*** is the weighted average of PM infected leaf area % at the sapling level, ***SR*** is the species richness gradient (1–4), ***DR*** is the three-level rainfall reduction gradient and ***SP*** is the absence/presence in the local neighbourhood of the five FORBIO three species. Similar models were run testing the influence of neighbourhood species composition on PM mycophagy, but including a covariate for PM levels as the potential for mycophagy could be expected to be greater if more PM was available. This covariate was always centered around its mean to improve interpretability of the parameter estimates.

The influence of composition and reduced rainfall on ladybird counts was modeled as a Generalized Linear Mixed Model using *lme4*, assuming a Poisson distribution. A negative binomial distribution was assumed if overdispersion was suspected, as indicated by a χ^2^ test on the ratio between the residual deviance and degrees of freedom using the function found at http://glmm.wikidot.com/faq/. The high frequency of zeroes and stochasticity of the data due to (adult) ladybird mobility lead us to sum the counts across all four measuring moments. Larvae were already very rare at this time of year and were treated as adults when summing the data.

### Lab experiment

In December 2014, 21 oak saplings were planted in pots of 15 L and top diameter of 32 cm, using potting soil with N-P-K content of 14-16-18 and 13% organic matter. The saplings were already in hibernation and had faced subzero temperatures before being removed from the field. All were placed in a climate chamber with 15 h of light and 9 h dark at constant temperature of 21°C. Each pot received weekly watering of 1 L, corresponding to a monthly precipitation level of about 56 mm. Monthly summer precipitation (June–September) averaged 73 mm in Zedelgem in the last 8 years, with 56 mm being the low end of the 95% confidence interval (KMI, Belgium). The lower estimate was chosen as poorer drainage was expected in the lab conditions.

Half January the first leaves started to appear. Three older oak saplings infected with PM had been removed from the field in late summer and were kept in the climate chamber to serve as inoculation for the other saplings. These older saplings still had fresh leaves and live PM mycelia by the time the last of the 21 saplings started to leaf out. One month after initial leafing out, all trees had fully developed their leaves and all were infected with PM. On from this point, two groups of 7 saplings received only 0.8 L and 0.6 L of water per week respectively, mimicking the reduced rainfall treatment used in the field. Saplings were sorted in the different watering treatment groups (i.e. 1, 0.8 and 0.6 L per week) in a random manner. Soil moisture content by volume was measured from the topsoil two weeks, six weeks and ten weeks after watering treatment began, using a ML3 ThetaProbe (Delta-T Devices) set with the standard conversion parameters for an organic soil. The crown area infected by PM was also evaluated three times: three, six and nine weeks after the watering treatment began. The protocol used was similar to the one used in the field, but because of the smaller crowns and lower amount of trees, classes of 10% were used.

**Fig 3:**
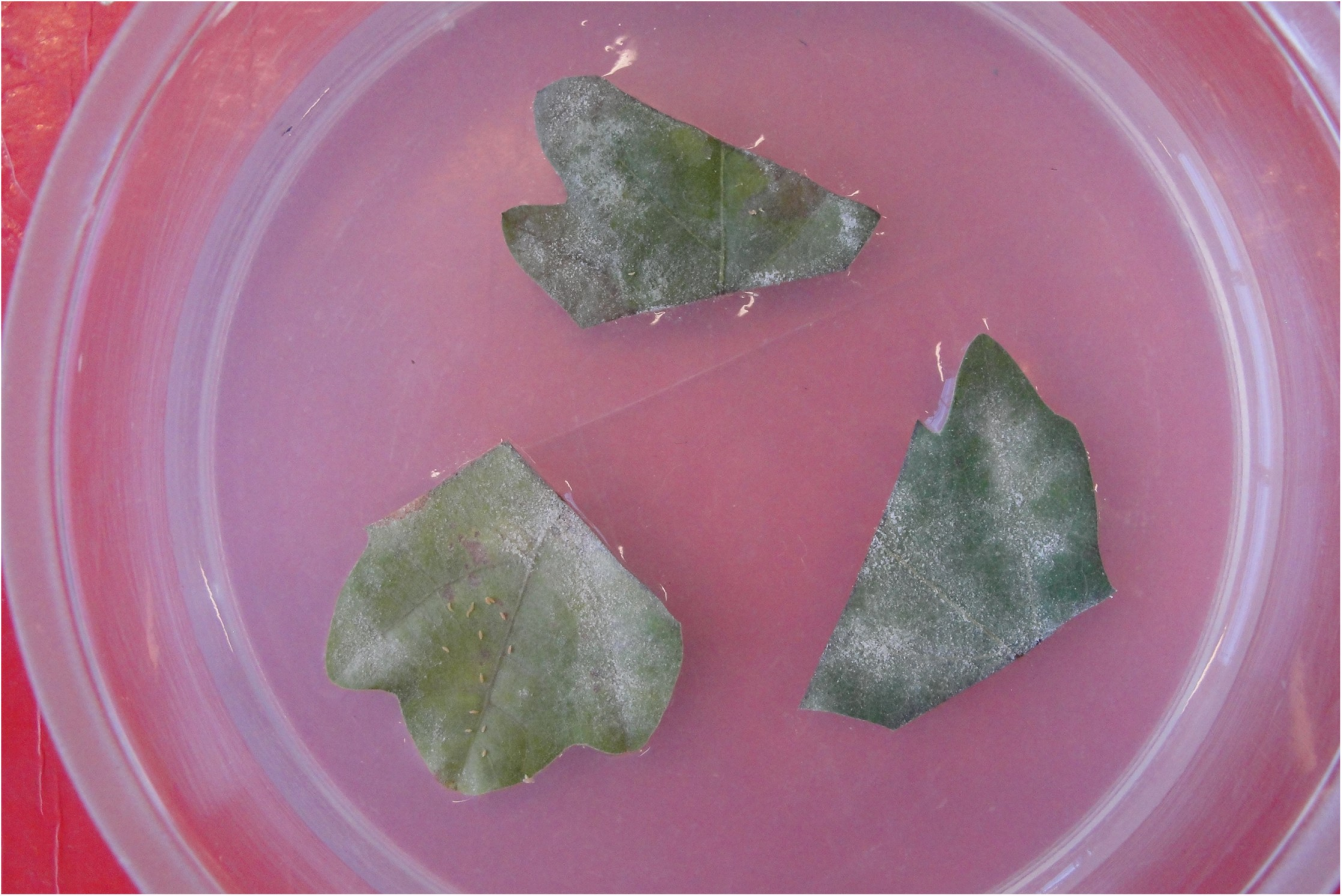
Example of a ladybird choice feeding experiment. Picture taken after 24 hours. The ladybird was removed to take this picture, but evidence of mycophagy can be noted in particular on the two leaf segments on the left. The leaf on the bottom also contains multiple particles of feces.

In late April and early May, four experiments on mycophagy by *P*. *vigintiduopunctata* were performed using leaves from the 21 saplings. Leaves of four saplings of each watering treatment were harvested and cut into segments that could fit in a Petri dish (Fig 3). Leaves were picked to minimize differences in PM infected area and leaf age (as lammas shoots had already formed in most saplings) between the watering treatments. Three leaf segments of the different watering treatments were placed on agar substrate (Micro Agar M1002) in a Petri dish with a ventilation hole (Fig 3). An adult *P*. *vigintiduopunctata* was placed at equal distance from the three segments and monitored for mycophagy with a handheld camera every 24 hours, including an initial snapshot and lasting up to 96 hours by which time leaves started to show signs of wilting. Ladybirds were removed as pictures were taken and reintroduced afterwards at their initial starting point. In each of the four experiments, four male and four female ladybirds were monitored. The male and female group each received leaf segments originating from the same 12 saplings. The ladybirds used for the experiment were adults who had been captured in the autumn of 2014 and kept fed in a climate chamber (16/8 light and dark treatment and constant temperature of 20°C) with fresh PM-infected leaves of *Q*. *robur*, *Convolvulus sp*. and *Sonchus oleraceus*. Before the feeding experiment, these ladybirds were subjected to a four month hibernation treatment of 4°C to ensure they would not be in diapause. Mycophagy was quantified using image analysis with APS Assess 2.0 (Lamari 2008). For each image, the PM infected area was digitally selected by modifying saturation. Differences in infected area were defined as mycophagy.

The influence of the water treatment was assessed in *lme4* [40] using a repeated measures mixed model, with the four different experiments and the ladybird used as random factors:

MP=DR+T+G+PM+DR*T+DR*G+T*G (3)

Where MP is the mycophagy in cm^2^, DR is the three-level watering treatment, T is the time of measurement (24-48-72-96h), G is gender of the ladybird and PM is initial PM affected area in cm^2^, centered around its mean. We also tested separately using a General Linear Model whether there was any difference in initial PM affected area between the three watering treatments.

## Results

### Field experiment

**Fig 4:**
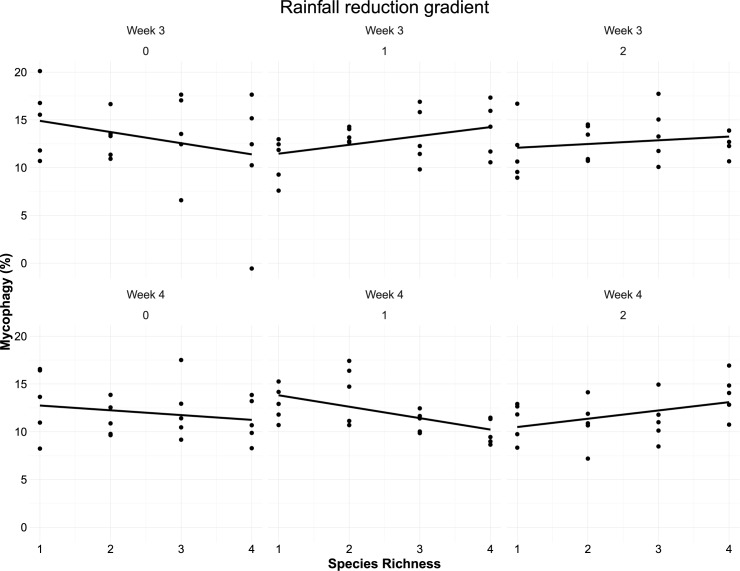
PM mycophagy in function of species richness in weeks 3 and 4 of monitoring, when a significant interaction was found. The mycophagy scores were transformed according to the effect of the PM covariate.

We found no influence of reduced rainfall or of species richness on PM infection rates (Table 1). We did see consistently higher PM rates if *Tilia* was present in the neighbourhood, but only for the phytometers receiving reduced rainfall (S1 Table). PM was about 10% lower in the last two weeks than it was in the first two. In the last two weeks, we found an interaction between species richness and reduced rainfall impacting mycophagy (Fig 4). Mycophagy overall did not change if rainfall was more strongly reduced (Fig 4), but the impact of neighbourhood species richness increased–resulting in less mycophagy in monocultures but more in mixtures when comparing control phytometers to those receiving less rainfall. As expected, phytometers with greater PM infected area also showed greater mycophagy (Table 1). In the presence of *Fagus*, mycophagy was reduced but only on phytometers under the control treatment (S1 Table).

**Table 1:** Parameter estimates of the mixed models of the PM and mycophagy scores (%) and total number of ladybirds over 4 measuring moments in function of rainfall reduction (DR) and species richness (SR).

		DR1	DR2	SR	DR1*SR	DR2*SR	PM.c (%)	C		
**PM**	Week 1	0.5	-5.86	0.92	-0.24	1.6		29.13**		
	Week 2	-0.48	-9.15	0.67	0.78	2.76		32.39***		
	Week 3	6.47	-0.64	2.79	-2.36	-1.15		17.76***		
	Week 4	0.48	-3.8	-0.2	0.66	1.07		22.03***		
**Mycophagy**	Week 1	-1.94	-1.77	-0.16	0.49	1.29	0.4***	16.82***		
	Week 2	-4.03	0.73	-0.44	1.73	0.05	0.36***	15.84***		
	Week 3	-5.51*	**-4.38°**	**-1.16°**	2.09*	**1.55°**	0.39***	16.03***		
	Week 4	1.78	**-3.63°**	-0.5	-0.69	1.37*	0.38***	13.22***		
**Ladybirds**	Poisson	0.08	0.75**	0.03	0.07	-0.15		1.62***	Ratio = 1.74***	
	Negat Binom	0	0.72	0.02	0.1	-0.15		1.68***		

C is the intercept, PM.c is the PM score covariate centered around its mean when applicable. Statistical significance is indicated using °, *, ** and *** indicating p < 0.1, 0.05, 0.01 and 0.001 respectively. Overdispersion was present for the ladybird count model as indicated by the ratio and its χ^2^ test.

We found no significant influence of reduced rainfall or composition on ladybird occurrence. There were generally more ladybirds (larvae + adults) on the phytometers receiving less rainfall (121, 158 and 176 total over 4 weeks respectively), but this effect was not significant when using a negative binomial distribution instead of Poisson after overdispersion was found (Table 1). Across the four monitoring sessions, we found no pupae and only few larvae of which only 1 in the control trees, compared to more than 10 on the trees receiving less rainfall. An identity model for ladybird count data yielded no significant effects, but the high complexity of the mixed model also resulted in a lack of convergence.

### Lab experiment

**Fig 5:**
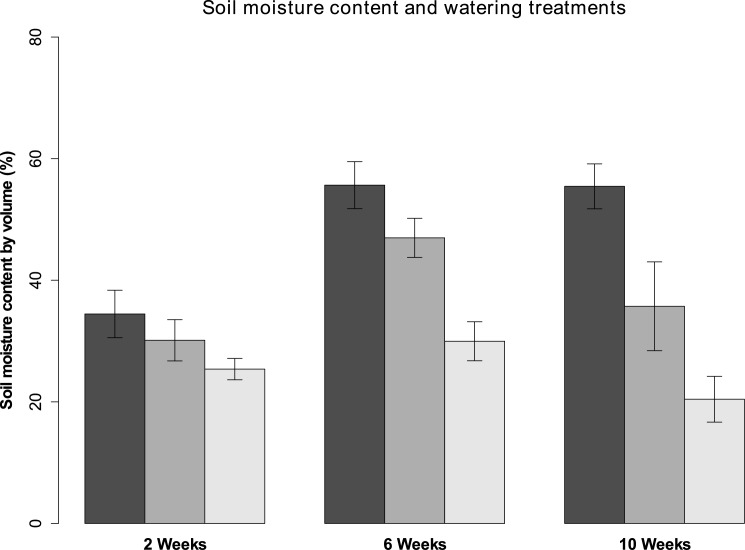
Soil moisture content by volume of the lab experiment oak trees. The different watering treatments (black = 1L, dark grey = 0.8 L and light grey 0.6 L per week) are shown with their 95% confidence intervals.

**Fig 6:**
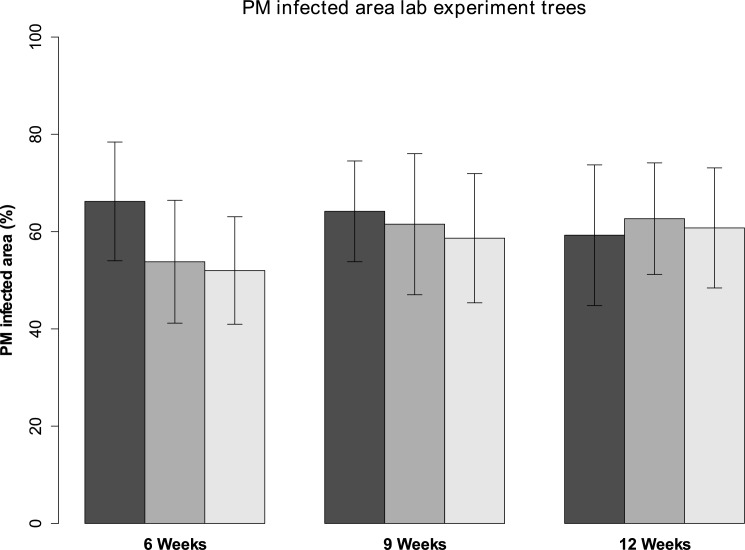
Average PM infected area of the lab experiment oak trees. The different watering treatments (black = 1L, dark grey = 0.8 L and light grey 0.6 L per week) are shown with their 95% confidence intervals.

The difference in soil moisture content between the three watering treatments continued to grow as the experiment ran and was significant (p < 0.01 in one-way ANOVA) before the choice feeding experiments began (Fig 5). The moisture content values were all rather high, rarely dropping below 20%. There was a small, insignificant initial difference in PM infected area between the trees receiving less water and the control (p = 0.06 in one-way ANOVA), but this was not found again during later measurements (Fig 6).

**Fig 7:**
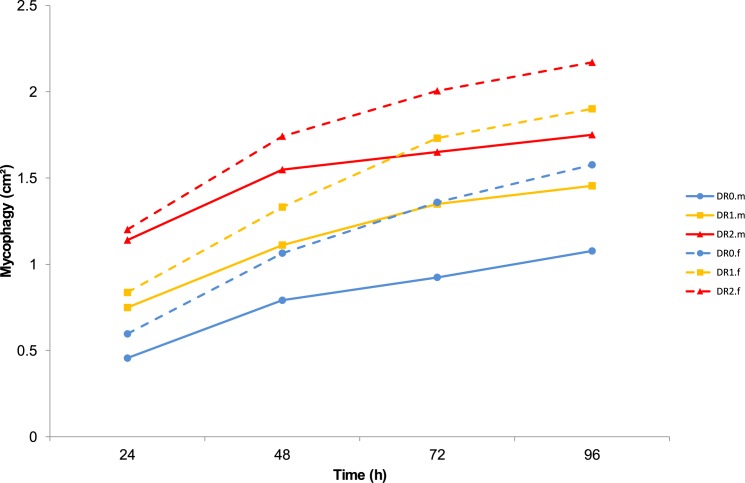
Mycophagy in function of choice experiment running time. The different watering treatments are distinguished (DR0, 1 and 2 corresponding to weekly watering of 1, 0.8 and 0.6 L respectively) and ladybird gender (m = male, f = female). Values were determined based on fixed parameter estimates of the full mixed model at average initial PM infected area.

In the choice feeding experiment, we found that PM infected leaves from trees that received less water were significantly more grazed upon than the control (Fig 7). The effect followed the gradient we applied, mycophagy increasing by 0.33 and 0.67 cm^2^ for the 0.8 and 0.6 L treatments respectively (p < 0.01 in the full model; extensive model results can be found in S2 Table). The overall average PM consumption after 96 hours was 1.54 cm^2^ per leaf segment and 4.61 cm^2^ per ladybird. This represents on average 51% of the PM on each leaf segment and 50% of all PM available for each ladybird. Complete removal by grazing of all PM on a single leaf was rare and not related to watering treatment, though it did not occur at all on the control leaves (data not shown). After 24 hours, males removed 1.95 cm^2^ and females 2.31 cm^2^ on average. Thus, females appeared to graze more than males and the difference increased with time (Fig 7), but this difference was not significant in our model (p = 0.18). While initial PM area positively influenced mycophagy (p = 0.001), it did not differ significantly between the three watering treatments (p = 0.94, one-way ANOVA). There were no significant interactions between the watering treatment (S2 Table), gender and running time of the experiment, though females did seem to keep up more steady mycophagy rates than males (Fig 7).

## Discussion

In our field experiment, we found effects of increasing species richness on mycophagy becoming stronger as more rainfall was removed along a stress gradient–corresponding to expectations of interplay between biodiversity effects and the Stress Gradient Hypothesis. This can be seen as evidence of an associational resistance effect through the actions of natural enemies, modulated by an environmental variable. However, we did not find an effect of species richness on ladybird counts. Hence, this would imply a mechanism by which greater species richness renders PM fungi more vulnerable, but not more attractive, to mycophagous ladybirds and only if the fungus is also (directly or indirectly) affected by lower plant water availability. This effect was apparently too small to have an impact on initial PM levels–as indicated by the lack of an effect of species richness or our reduced rainfall gradient on PM infected leaf area.

The lack of an effect on initial PM levels in our field study might be explained by the higher than average precipitation in August 2014 (112 mm, compared to a 1981–2010 monthly average of 76 mm), which would make mechanisms through plant water status less clear. September 2014 was extremely dry (8 mm compared to a monthly average of 76 mm) and only then did we find an interactive effect of reduced rainfall and neighbourhood species richness. As we scored PM infected area to include freshly grazed mycelia, it is likely that our initial (total) PM scores corresponded to those of the previous wet month. An alternative possibility is that mycelia loss due to mycophagy is swiftly replaced by the fungus. This would be supported by the results ofDillen et al. [17], where still no interaction between species richness and the reduced rainfall gradient on levels of PM was found for the very dry month of August 2013. If this is the case, the impact of mycophagy on PM may be limited. Previous studies where ladybird PM mycophagy was quantified focused on short-term removal rates, mostly under laboratory conditions [24,27]. However, in a study of mycophagous mites on grape PM, Melidossian et al. [42] did note a significant trophic impact on the disease over a three-year period, but they specified that this effect was strongest if biological control was possible at the early stages of PM infection–whereas our measurements occurred later in the growing season.

In the lab choice feeding experiment, a higher preference of ladybirds for PM on infected leaves originating from trees that received less water was found. Our PM removal rates after 24 hours and the gender difference were consistent with results in another study using the same ladybird species but other PM species and other environmental conditions [27]. Similar to the field experiment, we found no impact of watering rate on PM infected area, furthermore suggesting that the mechanism of different watering rates does not impact PM infections directly, only their being grazed upon by ladybirds. Possibly, a reduced access to water may impair the fungus’s defenses against mycophagy or increase its nutritional quality for the ladybird. While oak PM is known as a xerophilic pathogen [15], this is largely because of its tolerance of dry meteorological conditions and vulnerability to the physical effects of precipitation–which were not altered by our stress gradient. Pap et al. [36]found germination of PM conidia even at very dry levels of air humidity (down to 10%), but they also noted that high levels of humidity (90%) were associated with maximal germ tube length.

Another possibility is that plant water levels influence plant hormonal pathways that attract ladybirds to PM infected trees or reinforce resistance to fungal infection.Tabata et al. [37] noted strong attraction of *Psyllobora* ladybirds to 1-octen-3-ol, a Volatile Organic Compound (VOC). This alcoholic VOC is well-known as a fungal metabolite of the lipoxygenase (LOX) pathway, producing a moldy odor. However, in a study system of aphids, *Arabidopsis* and water stress, Truong et al. [43] found greater emissions of this and other alcohols under water stress and in the absence of aphid activity, while Kigathi et al. [44] noted 1-octen-3-ol as an Herbivore Induced Plant Volatile (HIPV) produced in certain (non-woody) Fabaceae species, possibly as a defense compound against herbivory. There is no known evidence of 1-octen-3-ol production in *Quercus*, other than as a result of fungal infections such as in cork from *Q*. *suber* [45]. Copolovici et al. [46]noted that PM-infected *Q*. *robur* leaves emitted less isoprene and more LOX products, which were chemically similar but not identical to 1-octen-3-ol. In another experiment with *Q*. *rubra*, higher emissions of LOX products were associated with heat stress [47]. However, they also noted that it is very difficult in practice to distinguish between emissions originating from the plant and from the fungus. Still, even if there is no endogenous 1-octen-3-ol production by the oak, changing water conditions inside the leaf might yet influence the VOC production by the fungus. As the hormonal pathways underlying the plant responses to different stressors interact [48,49], it would not be surprising to find a (positive or negative) impact of the leaf response to changing conditions of watering on its suitability for PM infection, which in turn could influence the palatability of the fungus for its fungivores. Such interactions between pathogens and water conditions may have implications in the light of future environmental changes associated with increased droughts, such as Climate Change, but more research into the underlying pathways is needed to offer more confident predictions.

## Conclusion

In this study, we found evidence of mitigating effects of species richness on oak PM, being modulated by environmental factors. These modulated mitigating effects were linked to multitrophic interactions with a mycophagous ladybird, which seemed to prefer feeding on infected leaves from trees with lower water status under laboratory conditions. These findings are in line with expectations of how the Stress Gradient Hypothesis interacts with positive effects of species richness on ecosystem functioning, in this case the mitigation of pathogen infection severity through trophic control. Previous studies have found little response of PM infections to dry or wet meteorological conditions, other than physical inhibition of spore germination by rain drops, and conflicting results for the effects of neighbourhood tree species richness on PM infections, but our results suggest that the same may not hold for ladybird mycophagous activity. Interactions between plant hormonal responses to abiotic and biotic stressors may explain this environmental modulation of trophic control, but more research is needed to elucidate these mechanisms. The substantial mycophagy rates we noted in both our field and lab study suggest that these mycophagous ladybirds may play a considerable role in mitigating the impact of oak powdery mildew. While we did not find a significant relationship between species composition and PM levels, our results suggest that mixing tree species may have a mitigating effect through enhanced ladybird activity under drier climatic conditions. In addition, given our observations in the field and during our lab study, promoting the presence of plant species vulnerable to other powdery mildew species, such as *Heracleum sphondylium* and *Sonchus oleraceus*, may strengthen ladybird activity by increasing their numbers and allow them to mix their diet.

## Supporting information

S1 Data Data (including metadata) collected in this study. (XLSX)

S1 Table Parameter estimates for the identity models of the field experiment. (DOCX)

S2 Table Results of the full model for the choice feeding experiment. (DOCX)
