# Supplementary material for: Stronger diversity effects with increased environmental stress: A study of multitrophic interactions between oak, powdery mildew and ladybirds
Source: PLoS One. 2017 Apr 18;12(4):e0176104. doi: 10.1371/journal.pone.0176104 (PMC5395233; doi:10.1371/journal.pone.0176104)
Supplement: S1 Table — (DOCX) [file pone.0176104.s002.docx]

S1 Table: Parameter estimates for the identity models of the field experiment. MP is mycophagy and PM is powdery mildew crown area %. Qu, Fa, Ti, Be and Pi represent respectively Quercus, Fagus, Tilia, Betula and Pinus presence in the local neighbourhood and C is the intercept. Interactions with the rainfall reduction (DR) treatment are indicated by 1 (medium) and 2 (severe reduction). Statistical significance is indicated using °, *, ** and *** indicating p < 0.1, 0.05, 0.01 and 0.001 respectively. All response variables were square root transformed due to residual heterogeneity.

|  |  | DR1 | DR2 | Qu | Fa | Ti | Be | Pi | PM.c | Qu1 | Qu2 | Fa1 | Fa2 | Ti1 | Ti2 | Be1 | Be2 | Pi1 | Pi2 | C |
| --- | --- | --- | --- | --- | --- | --- | --- | --- | --- | --- | --- | --- | --- | --- | --- | --- | --- | --- | --- | --- |
|  | Week 1 | -0.63 | -0.79 | 0.68 | 0.79 | -0.44 | -0.44 | -0.05 |  | -0.46 | 0.13 | -0.09 | -0.71 | **1.78°** | 1.10 | 0.44 | 0.46 | -0.89 | -0.10 | **5.32***** |
|  | Week 2 | -0.20 | -0.88 | 0.68 | 0.08 | -0.27 | -0.26 | 0.10 |  | -0.42 | -0.05 | -0.19 | -0.76 | **1.43°** | 1.11 | 0.82 | 1.37 | -1.29 | -0.64 | **5.59***** |
| PM | Week 3 | 0.58 | -0.04 | 0.87 | 0.41 | -0.16 | -0.06 | 0.56 |  | -1.22 | -0.93 | -0.78 | -1.28 | 1.15 | 1.18 | 0.78 | 0.71 | **-1.44°** | -0.79 | **4.11***** |
|  | Week 4 | -0.04 | -0.47 | 0.40 | 0.38 | -0.70 | -0.11 | 0.17 |  | -0.28 | -0.18 | -0.54 | -0.55 | **1.30*** | **1.43*** | 0.80 | 0.42 | **-1.16°** | -0.72 | **4.58***** |
| MP | Week 1 | -0.53 | -0.31 | 0.17 | -0.29 | 0.00 | -0.08 | -0.03 | **0.05***** | 0.24 | 0.19 | 0.23 | 0.42 | -0.13 | 0.37 | 0.26 | -0.15 | 0.14 | 0.04 | **4.12***** |
|  | Week 2 | -0.34 | 0.19 | 0.34 | **-0.58*** | 0.21 | 0.14 | **-0.46°** | **0.05***** | 0.04 | -0.33 | 0.43 | **0.62°** | -0.38 | -0.23 | -0.11 | -0.50 | **0.88*** | 0.49 | **3.85***** |
|  | Week 3 | **-0.71°** | **-0.75*** | -0.12 | **-0.67**** | -0.18 | **0.43*** | **-0.41°** | **0.06***** | **0.58°** | 0.32 | **0.67*** | **0.69*** | 0.10 | 0.50 | -0.32 | -0.27 | 0.45 | 0.19 | **3.95***** |
|  | Week 4 | 0.23 | **-0.56°** | 0.21 | **-0.52**** | -0.03 | 0.20 | **-0.31°** | **0.07***** | **-0.44°** | 0.11 | **0.41°** | **0.72**** | -0.26 | -0.10 | **-0.44°** | -0.06 | 0.27 | **0.49*** | **3.65***** |
