# Supplementary material for: Stronger diversity effects with increased environmental stress: A study of multitrophic interactions between oak, powdery mildew and ladybirds
Source: PLoS One. 2017 Apr 18;12(4):e0176104. doi: 10.1371/journal.pone.0176104 (PMC5395233; doi:10.1371/journal.pone.0176104)
Supplement: S2 Table — (DOCX) [file pone.0176104.s003.docx]

S2 Table: Results of the full model for the choice feeding experiment.

|  | Estimate | Std. Error | df | t value | Pr(>\|t\|) |  |
| --- | --- | --- | --- | --- | --- | --- |
| (Intercept) | 0.34 | 0.25299 | 23.5 | 1.327 | 0.19729 |  |
| t48h | 0.34 | 0.24552 | 334.7 | 1.366 | 0.1729 |  |
| t72h | 0.47 | 0.24552 | 334.7 | 1.907 | 0.05735 | . |
| t96h | 0.62 | 0.24552 | 334.7 | 2.53 | 0.01186 | * |
| DR1 | 0.29 | 0.23872 | 336.3 | 1.228 | 0.22039 |  |
| DR2 | 0.68 | 0.23794 | 335.1 | 2.877 | 0.00427 | ** |
| gender.female | 0.14 | 0.28931 | 84.2 | 0.486 | 0.62835 |  |
| PM (centered) | 0.12 | 0.03654 | 235.2 | 3.309 | 0.00108 | ** |
| t48h:DR1 | 0.03 | 0.30069 | 334.7 | 0.089 | 0.92886 |  |
| t72h:DR1 | 0.13 | 0.30069 | 334.7 | 0.438 | 0.66185 |  |
| t96h:DR1 | 0.08 | 0.30069 | 334.7 | 0.281 | 0.77873 |  |
| t48h:DR2 | 0.07 | 0.30069 | 334.7 | 0.243 | 0.80844 |  |
| t72h:DR2 | 0.04 | 0.30069 | 334.7 | 0.138 | 0.89004 |  |
| t96h:DR2 | -0 | 0.30069 | 334.7 | -0.035 | 0.97194 |  |
| t48h:gender.f | 0.13 | 0.24552 | 334.7 | 0.54 | 0.5893 |  |
| t72h:gender.f | 0.29 | 0.24552 | 334.7 | 1.199 | 0.23118 |  |
| t96h:gender.f | 0.36 | 0.24552 | 334.7 | 1.463 | 0.1445 |  |
| DR1:gender.f | -0.1 | 0.21541 | 339.5 | -0.246 | 0.80554 |  |
| DR2:gender.f | -0.1 | 0.21386 | 336.9 | -0.372 | 0.71007 |  |
